# The Comparison of Proteins Elaborated by *Streptococcus mutans *Strains Isolated from Caries Free and Susceptible Subjects

**Published:** 2013-04

**Authors:** Arezoo Tahmourespour, Abdolreza Nabinejad, Hannaneh Shirian, Nafiseh Ghasemipero

**Affiliations:** 1 Khorasgan-Isfahan Branch, Islamic Azad University, Isfahan, Iran; 2 Razi vaccine & serum Research Institute, Isfahan Branch (Vet Dept of Agriculture), Amirhamzeh, Isfahan, Iran; 3 Biotechnology Research Lab, Khorasgan-Isfahan branch, Islamic Azad University, Isfahan, Iran; 4 Lab Instructor of Microbiology, Khorasgan-Isfahan Branch, Islamic Azad University, Isfahan, Iran

**Keywords:** Caries free, Caries susceptible, Protein pattern, SDS-PAGE, Streptococcus mutans

## Abstract

***Objective(s):*** The oral streptococci especially mutans Streptococci are related with the development of caries in humans. However, they are commonly distributed not only in populations with moderate or high caries incidence but also in populations having no or low caries experience. So, in this study, the differences between protein profiles of *Streptococcus mutans* from different sources were compared.

***Materials and Methods:*** Twelve* S. mutans* strains were isolated from caries-susceptible, caries-free subjects and also *S. mutans*ATCC35668. Total proteins of the strains were extracted, and were analyzed through SDS-PAGE technique with coomassie staining.

***Results:*** The protein profiles of caries-susceptible subjects were different from those of caries-free subjects. The major protein bands of SDS-PAGE analysis were observed 26-100kDa and 45-57kDa in caries-susceptible and caries-free subjects, respectively. The major protein bands of SDS-PAGE analysis of *S.mutans *ATCC35668 were between 35-100kDa. Major significant differences between the protein patterns of two subject groups and interestingly, non-significant differences between the strains of each group were also found. The significant differences between the protein band number of S.mutansATCC35668 and caries-free isolates were also observed; while, there were no significant differences between *S.mutans*ATCC35668 and caries-susceptible subject isolates. The analysis of clusters by Hierarchical cluster method confirmed the clear distinction between *S.mutans* strains of two groups. Also, there was a clear distinction between the strain of *S.mutans*ATCC35668 and caries free strains

***Conclusion:*** The less protein bands and diversity in caries-free rather than caries-susceptible isolates may be the less cariogenicity it may cause. It is also revealed that the protein pattern analysis of S.mutans strains can be the suitable way for differentiation of them.

## Introduction

Mutans streptococci are the normal flora of oral cavity, but they can cause dental caries and periodontal diseases in many cases. There has been considerable interest in the *Streptococcus mutans* strains that are considered the main etiological agent of dental caries in humans (1-4). There has been a trend to group these organisms on the basis of their biochemical activity into the species *Streptococcus mutans*. However, the differences in the immunologic, morphologic and deoxyribonucleic acid (DNA) characteristics have been reported for this species (5). Molecular techniques are major tools for the characterization of bacteria from food and other biological substances. Sodium dodecyl sulfate-polyacrylamide gel electrophoresis (SDS-PAGE), DNA Amplification Finger Printing (DAF) and Restriction Fragment Length Polymorphism (RFLP) are the molecular techniques used for the characterization of bacterial macromolecules and are of significance importance (6). SDS-PAGE is an important molecular technique used for the identification at species level of whole cell proteins and it has the advantage of being fairly simple and rapid to perform, also in most proteins, the binding of SDS to the polypeptide chain imparts an even distribution of charge per unit mass, thereby resulting in a fractionation by approximate size during electrophoresis (7, 8). So, the SDS-PAGE gel electrophoresis is performed in order to measure the molecular weight of the protein subunits. (9-11).

The electrophoretic protein pattern of *Streptococcus mutans* strains for their differentiation into two groups with high and low cariogenicity potential has not yet been reported. 

The objectives of the present study are to explore the applicability of SDS-PAGE techniques for differentiating the electrophoretic profiles of the proteins elaborated by strains of *S.mutans* isolated from caries-free and caries-susceptible subjects and the comparison of protein profiles between each group and also with Standard Strain S. mutans ATCC35668. 

## Materials and Methods

*Subjects*

The study groups consisted of seven young adults with the mean age of 23 years. The group of caries-free individuals [DMF (Decayed, Missing, Filled) = 0] contained four subjects that successful isolation of *S. mutans* was carried out from two of them. The group of caries-susceptible individuals [DMF = 8 ± 2] contained five subjects, Standard strain of *S.mutans* ATCC35668 was also used for comparison.

*Sampling*

Volunteers were informed not to brush their teeth during the preceding 12 hr and not to drink or eat anything for 2 hr before sampling. Pooled samples of dental plaque were taken with sterile dental curettes from surfaces of the anterior and posterior teeth. 

*Culture*

The samples were cultured on Mitis Salivarius Agar (Merck, Germany) supplemented with 2% sucrose (Synth) and 0.2U Bacitracin ml^-1^ (Sigma) (12). Plates were incubated at 37 ºC for 48h in an atmosphere of 10% CO_2_ (13). The identification of strains in species level was done according to biochemical tests (sugar fermentation) and also confirmed with the help of Rap ID STR Kit (Remel co. Germany).

*Production of bacterial extracts*

In Order to obtain sufficient concentrations of proteins, 10^11^ cells of each isolate were grown in Brain Heart Infusion (BHI) broth for 18 h at 37 ºC with shaking (160 r.p.m.) in a shaker Incubator (Heidolph, Germany). Cells were collected by centrifugation (10000 × g for 15 min) and washed three times in 40 mM potassium phosphate (pH 7.5) with 3mM Dithiothreitol, 10 mM L. cysteine HCl, 0.06 mM MnSO4 (14). Cells are normally lysed by sonication (Hielscher, Germany) with microtip for 30 to 60 s, with ice-bath cooling. After lysis and centrifugation at 30,000 × g for 20 min, aliquots of the several milliliters of lysate (supernatant) were transferred to three or four culture tubes and stored at -20 ºC until used for electrophoresis***.*** Protein concentrations were also determined by the Lowry method (15).

*SDS-PAGE Electrophoresis*

Electrophoresis was performed in 10 % (w/v, polyacrylamide) resolving gels (1.5M Tris-HCl pH 8.9, 10% SDS, 10% acryl amide + bisacrylamide) and 2.5% stacking gels (0.5 M Tris-HCl pH 6.9, 10% SDS, 2.5% acryl amide +bis) by the method of Laemmli (1970) using 0.75 mm thick slab gels and Tris/HCl buffer (pH 6.8)(16). Samples were diluted in an equal volume of sample buffer (0.2 g SDS, 1 g sucrose, 500 µl β-mercaptoethanol, and 0.002g bromophenol blue, 1 M Tris /HCl) and boiled for 5 min at 95 ºC. A marker of known molecular weight (Fermentas SM 0661 protein ladder) was also loaded (20μl) along with the samples. The apparatus was connected with constant electric current (30mA) till the bromophenol blue (BPB) reached the bottom of the plate. After electrophoresis (120 min, 30 mA), the gels were placed into a container with staining solution-containing coomassie brilliant blue (CBR) R-250 dissolved in methanol (Merck, Germany) with acetic acid (Merck, Germany) and double distilled water. Gels were kept in the staining solution for overnight and destained in methanol, acetic acid and water with shaking (Heidolph, Germany) until the bands appeared. 

*Comparison of protein patterns*

The average similarity, S, between two strains was assessed, whereby average similarity = (number of matching bands × 2) /total number of bands in both strains. This calculation was based on a visual comparison of bands above 20 kDa. It was performed only between patterns on the same gel, because visual comparison did not allow the correction of gel-to-gel variations (17). All strains were run 5 times to confirm results. 

*Statistical analysis:*

The excel software was used for the comparison of means by standard error. Strains were also clustered according to their protein pattens by Hierarchical cluster method (SPSS16) (18). 

## Results

Twelve *Streptococcus mutans* isolates were isolated and analyzed (Table1). Six strains of them were isolated from caries-susceptible subject persons and, five strains were isolated from caries-free persons. 

**Figure 1:**
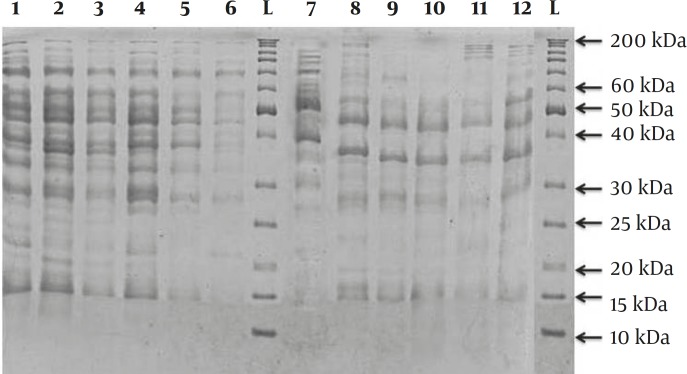
SDS-PAGE analysis of protein extracts of various Streptococcus mutans strains. Each well contained 3µg of proteins and the gel was staied with coomassie blue. The molecular size of some bands is indicated on the side of the figure

The SDS-PAGE of protein extracts of isolates showed between 6 to 18 bands with molecular masses in the range of 15-200 kDa (Figure 1). The major protein bands of SDS-PAGE analysis were observed 26-100 kDa and 45-57 kDa in caries-susceptible subject (C1, C2, D3, F4, F5 and G7) and caries-free -subjects (A8, B9, B11, P12 and P13), respectively; while, the major protein bands of SDS-PAGE analysis of standard strain *S.mutans* ATCC35668 were between 35-100kDa. The differences between the heavily stained bands were more frequent in two sample groups (caries susceptible subject and caries-free subjects). These heavily stained bands for caries susceptible subject (C1, C2, D3, F4, F5 and G7) were between the 28-70 kDa regions; while, the heavily stained bands for caries-free subjects (A8, B9, B11, P12 and P13) were between the 33-52 kDa regions. The reproducibility of SDS extracts was very high. 

**Table 1:** List and origin of used *Streptococcus mutans* strains

Pacient	Strain	Strain number	Origion
C	S.mutans C1	1	Dental plaque of caries-susceptible subject
C	S.mutans C2	2	Dental plaque of caries-susceptible subject
D	S.mutans D3	3	Dental plaque of caries-susceptible subject
F	S.mutans F4	4	Dental plaque of caries-susceptible subject
F	S.mutans F5	5	Dental plaque of caries-susceptible subject
G	S.mutans G7	6	Dental plaque of caries-susceptible subject
-	S.mutans ATCC35688	7	-
A	S.mutans A8	8	Dental plaque of caries-free subject
B	S.mutans B9	9	Dental plaque of caries-free subject
B	S.mutans B11	10	Dental plaque of caries-free subject
P	S.mutans P12	11	Dental plaque of caries-free subject
P	S.mutans P13	12	Dental plaque of caries-free subject

Each letter (A-P) stands for each volunteer; numbers are related to the strain number e.g. C1 indicates that strain number 1 is isolated from pacient

**Lane 1-6 is related to strains isolated from caries-susceptible subject including C1, C2, D3, F4, F5 and G7. Lane 7, S.mutans ATCC35668. Lane 8-12 is related to strains isolated from caries-free subjects including A8, B9, B11, P12 and P13**

**Figure 2:**
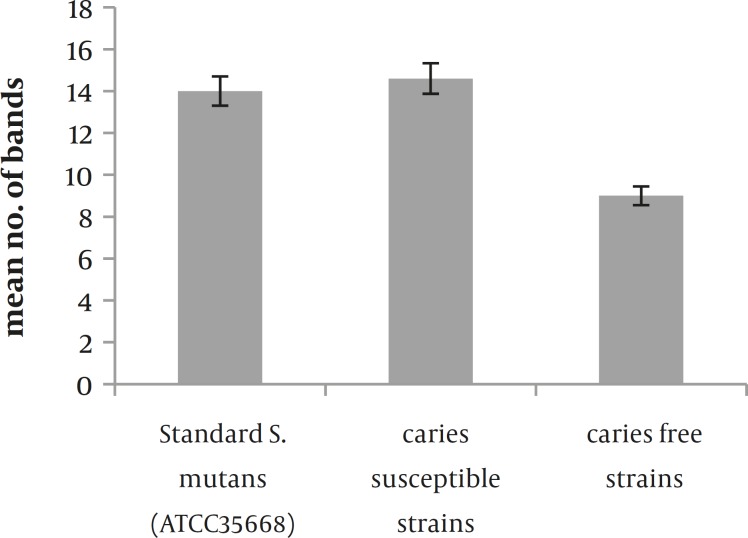
The comparison of mean band number between sample groups and standard strain resulted from SDS-PAGE analysis (significant differences are at 5% level);

**Figure 3:**
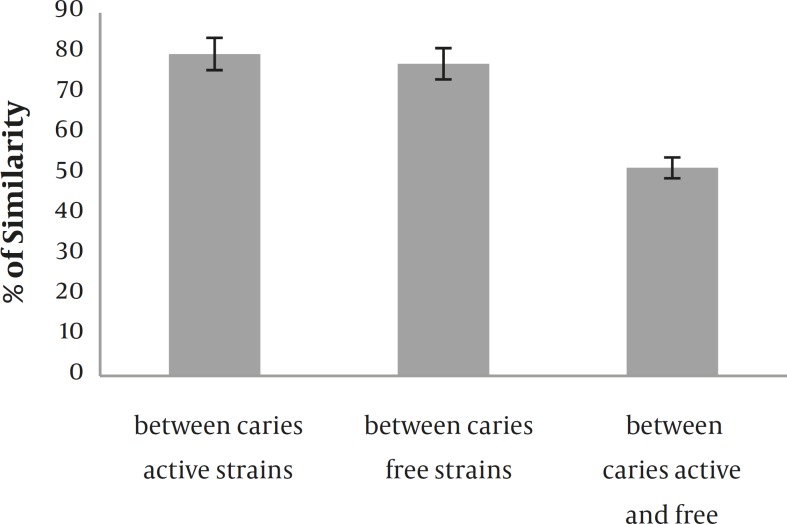
The average similarity, S, between study groups, whereby average similarity = (number of matching bands × 2) /total number of bands in both strains. This calculation is based on a visual comparison of bands above 20 kDa (significant differences are at 5% level).

**Figure 4:**
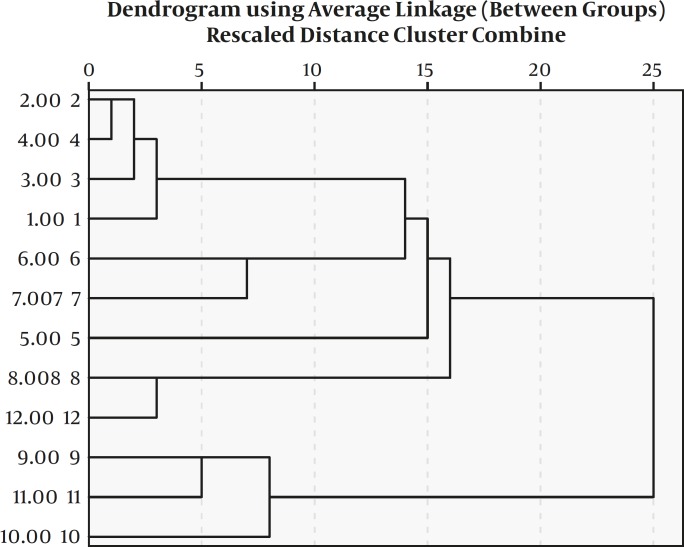
The clusters analysis of strains by Hierarchical cluster method

The protein nature of major bands was assessed by their colorations with Coomassie blue. SDS-extracted protein patterns were strictly specific for Strains of *S.mutans* and related to strains and the origin of isolates (Figure 1). Only a few major bands appeared in the lower part of the gel (22-15 kDa). Major differences could be seen between protein patterns of two subject groups and also standard strain. Interestingly, these differences between species were also found except in the case of C1 and C2, which were isolated from the same patient. They are considered as same clonal type due to 91.3% similarity, S, between SDS-PAGE protein patterns.

F4- F5 (S = 69 %), B9-B11 (S = 78.3 %) and P12-P13 (S = 65.2 %) were not considered as the same clonal type, although both of them were isolated from same person. It should be mentioned that F4 - F5 were isolated from one subject who was caries susceptible, while B9-B11 and P12-P13 were isolated from caries-free subjects.

According to Figure 2, it is clear that there is a significant difference between the numbers of protein bands appeared in sample groups (caries active and susceptible strains).Also there is a clear distinction between the strain of *S. mutans* ATCC35668 and caries free strains.

The representation of these differences was assessed by the calculation of the average similarity, with visual comparison (Figure 3). In spite of the visual comparison, good reproducibility was obtained because of the high resolution of gels. The analysis of clusters by Hierarchical cluster method confirmed the clear distinction between *S. mutans* strains of two subject groups (Figure 4). 

## Discussion

The results indicate that the electrophoretic techniques prove to be quite effective in providing information on the numbers, types, relative quantities, and electrophoretic characteristics of the proteins of *S. mutans* strains isolated from caries free and susceptible subjects. Since the total protein patterns of the present study are reproducible, they might be helpful in differentiating isolated strains of *S. mutans*. 

Past reports have shown that electrophoretic patterns of bacterial cell wall or intracellular proteins could serve such purposes (19, 20, and 7). Ogunjobi *et al* (2007) and Razaghi *et al* (2012) have shown the level of relatedness and diversity among the bacterial population (Xanthomonas sp.) by clustering the SDS protein banding pattern (21, 22). Abbasi *et al* has also demonstrated the existence of a considerable genetic diversity among *Pseudomonas *strains through this technique (23).

 The electrophoretic conditions used in this investigation are found to be optimal for our purposes after testing various combinations of the pH and acryl amide gel concentration . In this study, the differences of protein patterns of sample groups,caries-susceptible subject and caries-free subject, has been determined by SDS-PAGE analysis. Electrophoretic analysis of the protein extracts of whole *S. mutans* strains has revealed significant differences between sample groups (*P*value <0.05) and non-significant differences within the strains of each sample groups (*P*value >0.05). 

Several studies have also shown genetic heterogenicity among *S. mutans* starins by different methods (24, 25). However, there are controversial studies of *S. mutans* genetic diversity relationship with caries activity (24, 26). While Alaluusua *et al* (27) detected a greater genotypic diversity of cariogenic streptococci using ribotyping (27), Kreulen *et al* (1997) did not show the same results using AP-PCR technique in *S. mutans* isolates and suggested the less genetic diversity (28).

## Conclusion

The *Streptococcus mutans* isolates are genetically diverse. Therefore, the less protein bands and diversity are in caries-free rather than caries-susceptible isolates, the less cariogenicity it may cause. It is also revealed that the protein pattern analysis of *S. mutans* strains can be the suitable way for differentiation of them.
